# Phybrata Sensors and Machine Learning for Enhanced Neurophysiological Diagnosis and Treatment

**DOI:** 10.3390/s21217417

**Published:** 2021-11-08

**Authors:** Alex J. Hope, Utkarsh Vashisth, Matthew J. Parker, Andreas B. Ralston, Joshua M. Roper, John D. Ralston

**Affiliations:** 1 AltaML Inc., Edmonton, AB T5J 3N9, Canada; alex@altaml.com (A.J.H.); utkarsh.vashisth@live.in (U.V.); mj3parker@gmail.com (M.J.P.); 2 PROTXX Inc., Menlo Park, CA 94025, USA; andreas.ralston@protxx.com (A.B.R.); josh.roper@protxx.com (J.M.R.); 3 PROTXX Medical Ltd., 120, 4838 Richard Road SW, Calgary, AB T3E 6L1, Canada

**Keywords:** machine learning, wearable sensor, concussion, physiological impairment, vestibular, neurological

## Abstract

Concussion injuries remain a significant public health challenge. A significant unmet clinical need remains for tools that allow related physiological impairments and longer-term health risks to be identified earlier, better quantified, and more easily monitored over time. We address this challenge by combining a head-mounted wearable inertial motion unit (IMU)-based physiological vibration acceleration (“phybrata”) sensor and several candidate machine learning (ML) models. The performance of this solution is assessed for both binary classification of concussion patients and multiclass predictions of specific concussion-related neurophysiological impairments. Results are compared with previously reported approaches to ML-based concussion diagnostics. Using phybrata data from a previously reported concussion study population, four different machine learning models (Support Vector Machine, Random Forest Classifier, Extreme Gradient Boost, and Convolutional Neural Network) are first investigated for binary classification of the test population as healthy vs. concussion (Use Case 1). Results are compared for two different data preprocessing pipelines, Time-Series Averaging (TSA) and Non-Time-Series Feature Extraction (NTS). Next, the three best-performing NTS models are compared in terms of their multiclass prediction performance for specific concussion-related impairments: vestibular, neurological, both (Use Case 2). For Use Case 1, the NTS model approach outperformed the TSA approach, with the two best algorithms achieving an F1 score of 0.94. For Use Case 2, the NTS Random Forest model achieved the best performance in the testing set, with an F1 score of 0.90, and identified a wider range of relevant phybrata signal features that contributed to impairment classification compared with manual feature inspection and statistical data analysis. The overall classification performance achieved in the present work exceeds previously reported approaches to ML-based concussion diagnostics using other data sources and ML models. This study also demonstrates the first combination of a wearable IMU-based sensor and ML model that enables both binary classification of concussion patients and multiclass predictions of specific concussion-related neurophysiological impairments.

## 1. Introduction

### 1.1. Challenges in Concussion Diagnosis and Management

Concussion injuries present a public health challenge that impacts many different professional and amateur sports [1], military training and deployment [2,3], and large pediatric [4], elderly [5], and civilian [6] populations. A concussion can lead to disruptions that are widespread throughout the brain [1,7]. As a result, patients can suffer from impairments to multiple physiological systems in their bodies [7], including the central nervous system (CNS; brain and spinal cord), peripheral nervous system (PNS; somatic, autonomic), sensory systems (visual, vestibular, somatosensory), neurovascular system, and musculoskeletal system. If not properly diagnosed and treated, these impairments can persist for months or years and negatively impact many aspects of the patient’s health. Extensive studies over the past decade have also established the link between repetitive head impacts and long-term degenerative decline such as chronic traumatic encephalopathy (CTE) [8].

Current solutions for diagnosing the above multiple concussion-related impairments and monitoring each individual patient’s response to treatments and rehabilitation either (i) require multiple time-consuming tests carried out by multiple clinical specialists using expensive lab equipment [9,10,11,12], often with very long delays; or (ii) are limited to subjective observations and patient self-reporting using clinical scales with limited sensitivity to change and significant administrator-dependent variability [13,14]. The lack of easy-to-use tools for quantifying impairments to multiple physiological systems can lead to misdiagnosis [15,16] and poor treatment outcomes [17]. Similar challenges are also encountered in the management of patients with many other neurological conditions caused by injuries, disease, aging, or genetic disorders, including stroke [18], elderly frailty [19], multiple sclerosis (MS) [20], and Parkinson’s disease [21].

Many advanced clinical neurology tools can be used to assess concussion-induced impairments, including structural and functional magnetic resonance imaging (MRI, fMRI) [12,22,23,24], balance assessments using computerized dynamic posturography [9,25,26], instrumented gait analysis [10,27], and blood biomarkers [28], but the cost and complexity of these approaches limit their use to research environments. As a result, the vast majority of concussion diagnoses still rely on clinical examinations that evaluate symptoms, neurocognitive status and function, and physical behavior using a wide variety of clinical scales and behavioral tests [13,14,29]. These examinations have been shown to have limited sensitivity to change and significant administrator-dependent variability [13,14,29]. As a result, there is a significant unmet clinical need for tools that allow physiological impairments and longer-term health risks to be identified earlier and better quantified, as well as to allow treatments and rehabilitation protocols to be bettered tailored to each individual patient’s unique impairment profile. The solution described in the present study combines a wearable neurophysiological impairment sensor with machine learning (ML) algorithms to enable much easier-to-use and lower-cost precision patient assessments and concussion diagnoses that can be carried out in any doctor’s office or via remote patient monitoring.

### 1.2. Machine Learning and Wearable Motion Sensors in Concussion Management

Applications of machine learning to help automate concussion diagnoses and recovery monitoring have attracted significant research interest in recent years [30]. Data sources include structural and functional MRI [31], electroencephalography (EEG) [32,33,34,35,36], clinical scales [13,30,32,37,38,39], balance and vestibular diagnostic data [37], gait analysis [30,40], eye tracking [41], blood biomarkers [42], analysis of head impact data [43,44,45], and a variety of wearable physiological sensors [33]. Wearable sensors are of particular interest because of their low cost, ease of use, and compatibility with remote patient monitoring. Within the wide range of physiological metrics that can now be measured using wearable devices, assessments of balance and gait have the greatest utility for identifying the multiple physiological system impairments that can result from concussions [46] and other neurological conditions [47,48]. This utility stems from the fact that balance and gait can be disrupted by the widely distributed impact-induced disruptions in the brain, vestibular system, visual system, or spinal cord that can accompany concussion injuries. Involvement of the primary sensory integration brain structures (e.g., brainstem, cerebellum) results in impaired central integration of key sensory system inputs (visual, somatosensory, and vestibular) and impaired generation of motor control outputs that are critical to enabling balance control [49] and stable gait [50]. Visual and somatosensory inputs are further impaired by the visual system and spinal cord disruptions. Balance and gait impairments are also correlated with cognitive impairment [46,51]. However, very few concussion specialists and patients have access to gold-standard laboratory research tools such as computerized dynamic posturography [9,25,26,49] for assessing balance and instrumented gait analysis [10,27,50] for assessing gait. This limited access has generated significant research interest in the past decade in the use of wearable motion sensors for measuring concussion-related balance and gait impairments [52,53]. Wearable motion sensors have already shown promise for use in the development of concussion gait and balance biomarkers with sensitivity and reproducibility superior to clinical rating scales for concussions [52,53] and a wide spectrum of other neurological conditions [54,55,56,57]. ML classification of wearable motion sensor data thus represents one very promising path forward for improving upon existing clinical options for diagnosing and managing concussions.

### 1.3. Phybrata Sensing

We recently introduced physiological vibration acceleration (phybrata) sensing [58], a non-invasive balance and neurophysiological impairment assessment tool that utilizes a head-mounted accelerometer to detect the contributions of individual physiological systems to a phenomenon that is unique to a head-mounted sensor—the biomechanical stabilization of the head and eyes as the reference platform that the body relies on for balance and movement. The phybrata sensor attaches behind the ear using a small disposable adhesive and testing simply requires the patient to stand still for 20 s with their eyes open (Eo), and again for 20 s with their eyes closed (Ec). During this static balance testing, the phybrata sensor uses a microelectromechanical system (MEMS)-based inertial motion unit (IMU) to detect microscopic involuntary motions of the body, both normal motions characteristic of healthy individuals and pathological motions caused by physiological impairments that impact balance and postural stability. As we previously described in detail [58], the direct measurement of acceleration motions at the head allows the phybrata sensor to detect signal components with a much wider range of frequencies compared to motion sensors mounted elsewhere on the body, which in turn allows impairments to individual physiological systems (CNS, PNS, sensory, musculoskeletal) to be identified, quantified, and monitored using the unique biomechanical vibrational signature of each system, and reveals the sensory reweighting across multiple physiological systems that is triggered by impairment to any single physiological system. Although many wearable motion sensors contain both an accelerometer and a gyroscope, accelerometers are significantly more power-efficient than gyroscopes [59], and the use of a single accelerometer in the phybrata sensor makes it more practical for long-term activity monitoring [60,61]. Previous research has shown that a single tri-axial accelerometer mounted on the sternum is adequate for monitoring people’s locomotor activities [60,62,63].

The above impairment classification leverages previous spectral analyses of postural sway time series data in which distinct frequency bands are observed to correspond to specific mechanisms of postural control [64,65,66,67,68] for example >1 Hz (spinal reflexive loops, proprioception, multi-joint and muscle activity); 0.5–1 Hz (CNS participation, both cerebellar and cortical); 0.1–0.5 Hz (vestibular regulation); 0.02–0.1 Hz (visual regulation). The much wider spectral content of phybrata signals compared to motion sensors mounted elsewhere on the body also reveals the sensory reweighting across multiple physiological systems that is triggered by impairment to any single physiological system, as the postural control system dynamically regulates to reduce dependence upon impaired inputs [58]. Disruptions to these neuromotor and neurosensory systems accompany many neurological conditions, including concussions [58,69,70], and are closely tied to concussion symptoms [7,15]. Digital biomarkers derived from unique spatial–domain, time–domain, and frequency–domain features and sensory reweighting profiles detected in phybrata signals were shown to demonstrate well-defined normative values that distinguish between healthy and impaired individuals across a wide population base with no previous baseline measurement required [69]. For the specific case of concussion injuries, receiver operating characteristic (ROC) analyses of phybrata digital biomarkers have demonstrated classification of healthy vs. concussed individuals with sensitivity, specificity, accuracy, and area under the curve (AUC) all above 90%, as well as separate independent classification of neurological and vestibular impairments, also with sensitivity, specificity, accuracy, and AUC all above 90% [58].

### 1.4. Distinguishing Neurological vs. Vestibular Impairments

Distinguishing between head-impact-induced vestibular system disruptions and brain injuries is vital since the appropriate course of treatment and rehabilitation will typically be quite different. Damage to the vestibular balance organs, vestibular nerve, and multiple connected neurological circuits are associated with many cognitive and physical symptoms arising from concussions [71,72], and damage to the vestibular system is often the predominant driver of long-term symptoms [73]. A recent ML cluster analysis of concussion patients revealed the presence of two distinct groups, one with prominent vestibular disorders and another with no clear vestibular or balance problems [37]. However, many concussion patients are still managed uniformly, despite the nature of their injuries, in the hopes that the pertinent physiological impairments will be addressed. Treatment efficiency and patient outcomes can be significantly improved using a tool such as phybrata testing to identify, quantify, and track changes in impairments to specific physiological systems.

Current gold-standard solutions to achieve the above ROC diagnostic performance require multivariate composite models that combine data from multiple time-consuming and expensive tests such as computerized dynamic posturography, eye tracking, and neuro-cognitive assessments to generate more complex multimodal concussion biomarkers [32,35,74,75,76]. The above ROC analyses utilized extensive manual feature inspection and statistical data analysis to select features in the phybrata signals that are predictive of specific physiological impairments and were limited to digital biomarkers derived from Eo and Ec phybrata powers [58] to quantify and monitor the progression of these impairments. In this paper, we assess the ability of four different ML models to identify and rank the importance of a wider range of phybrata features and feature sets to further refine the derivation of concussion biomarkers and automate impairment classification.

### 1.5. Present Study

Phybrata datasets have previously been leveraged to develop and train a convolutional neural network (CNN) ML model that can be used for a wide range of analytical applications [77]. The unique spatial–domain, time–domain, and frequency–domain features and feature sets observed in phybrata signals allowed the CNN model to achieve high levels of classification performance following training with relatively small datasets [77]. In this paper, we explore opportunities to further enhance the diagnostic performance of the phybrata sensor using a wider range of ML models. Of particular interest in the current paper are lower computational complexity ML models that may allow impairment classification to be incorporated directly into the phybrata sensor. Such a wearable device, capable of cloud-independent classification and quantification of multiple physiological impairments, would greatly enhance remote patient monitoring for the management of many chronic medical conditions.

**Figure 1:**
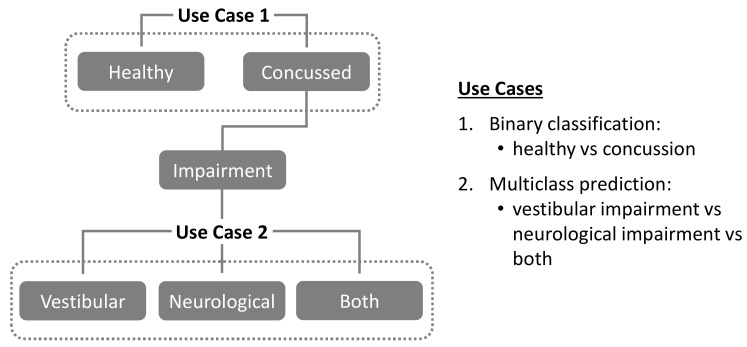
Classification use cases investigated in the present study.

The present study utilizes data from a previously reported concussion study population [58] to investigate the following two use cases, as illustrated in Figure 1:

Use Case 1. Binary classification of study population into “healthy” vs. “concussion” groups.

Use Case 2. For the above “concussion” group, multiclass prediction of specific physiological impairments (“vestibular” vs. “neurological” vs. “both”).

For Use Case 1, training, validation, and binary classification performance are evaluated and compared across four different ML models: Support Vector Machine (SVM), Random Forest Classifier (RF), Extreme Gradient Boost (XGB), and Convolutional Neural Networks (CNN). Further details and links to source code for the models themselves can be found in the Appendix A. In addition, two different data preprocessing pipelines are evaluated for each ML model: a) time-series averaging (TSA), and b) non-time series feature extraction (NTS). In Use Case 2, the best performing models from Use Case 1 are then evaluated for their ability to enable multi-class predictions of specific physiological system impairments (“vestibular” vs. “neurological” vs. “both”) in individuals diagnosed as “concussed”.

## 2. Materials and Methods

### 2.1. Study Population, Data Collection, Derivation of Phybrata Biomarkers

As we previously reported [58], data were analyzed from 175 patients at three clinical sites. Phybrata testing was included in regularly scheduled clinical patient assessments, the study was conducted in accordance with the Declaration of Helsinki under Western IRB Study Number 1,188,786, and informed consent was obtained for all participants in the study. Study participants included 94 females and 81 males (ages 18.1 ± 10.9 years, min 7 years, max 66 years), including 92 patients diagnosed with concussion (51 female, 41 male, ages 18.8 ± 13.2 years, min 7 years, max 74 years) and 83 healthy participants (43 female, 40 male, ages 17.2 ± 7.7 years, min 8 years, max 74 years). Comprehensive clinical concussion assessments were first completed for all patients, followed by testing with the phybrata sensor. Of the 92 patients diagnosed with concussion, 26 were diagnosed with vestibular impairments via clinical assessment, 40 with neurological impairments, and 26 with both vestibular and neurological impairments. A total of six patients with incomplete phybrata signal datasets were excluded from the ML analyses, leaving a sample size of 169 patients.

Patients were tested using the previously reported phybrata sensor [58,69] attached to the patient’s mastoid using a disposable medical adhesive while patients stood still for 20 s with Eo and then again for 20 s with Ec. During testing, participants were instructed to stand upright in a relaxed position with their feet together and their arms at their sides while maintaining their gaze in a straight-ahead direction. The phybrata sensor utilizes a 3-axis accelerometer to record *x* (anterior–posterior (A–P), or front–back), *y* (vertical), and *z* (medial–lateral (M–L), or left–right) acceleration time series data in units of g. During each 20 s test data are recorded at a sampling rate of 100 Hz, generating a total of 2000 samples for each of the 3 axes (*x*, *y*, *z*). The accelerometer signals are filtered to remove drift. Additional details of the sensor device, testing procedures, and data analysis were previously reported [58,69]. Phybrata time-series data and spatial scatter plots, Eo and Ec phybrata powers, average power (Eo + Ec)/2, Ec/Eo phybrata power ratio, time-resolved phybrata signal power spectral density (PSD) distributions, sensory reweighting profiles, and ROC curves were compared for individuals with no objective impairments and those clinically diagnosed with concussions and accompanying vestibular impairment, neurological impairment, or both vestibular and neurological impairments. In our previous work [58], manual feature extraction and ROC analyses indicated that the average power (Eo + Ec)/2 may be utilized to support clinical diagnosis of concussion, while Eo and Ec/Eo may be utilized as independent measures to confirm accompanying neurological and vestibular impairments, respectively. All three measures demonstrated AUC, sensitivity, specificity, and accuracy above 90% for their respective diagnoses.

### 2.2. Data Preprocessing

The following two preprocessing pipelines were utilized to prepare the phybrata time series data for the ML analysis in Use Cases 1 and 2:

1. Time-Series Averaging (TSA): For each Eo and Ec patient test phase, the three phybrata time-series signals (*x*, *y*, *z*) and the phybrata power (calculated using the vector sum of the three acceleration components [58,69]) were averaged over one-second time-steps (100 samples per step), reducing the dimensionality of each time series from 2000 samples to 20 samples. Once averaged, the data were either used in their existing form for CNNs or converted such that each time-step represents a column instead of a row for classical ML models. There are two reasons for using this averaging approach as an alternative to using the raw signal. First, the raw data contains 6000 measurements per patient test (100 Hz sampling over 20 s for each of the *x*, *y*, and *z* axes), which presents challenges for training classical ML models, since the number of data features greatly exceeds the number of patients. This excessive number of features can lead to models that overfit and generalize poorly to data from new patients. Second, the computational advantages in using an averaged time-series instead of a full time-series signal recording can enable much faster and lower computational complexity training and classification, allowing the use of remote sensor devices that do not require cloud connectivity for computational support. No frequency features were extracted from the TSA preprocessed data. Further details of the phybrata power calculations and data processing are included in the Appendix A.
2. Non-Time-Series (NTS) Feature Extraction: Standard statistical measures (variance, mean, standard deviation, min, max and median) were calculated for each of the three phybrata time-series signals (*x*, *y*, and *z* accelerations) and several additional power and frequency features extracted for both Eo and Ec test phases, including phybrata powers within the physiological-system-specific frequency bands discussed above. To extract the power features, the phybrata power was first calculated at each value in the accelerometer time-series data. The power values were then summed for each respective test phase (e.g., Eo Power and Ec Power) and the powers for the two phases were averaged (e.g., (Ec + Eo)/2). Phybrata signal PSD curves were also calculated using Welch’s method [78], and these PSD curves were then used to calculate phybrata powers within specific frequency bands. PSD variations within specific spectral bands, as well as correlated PSD variations across multiple spectral bands, were shown to help quantify the sensory reweighting that often accompanies many neurophysiological impairments [58] and may thus also serve as useful ML classification features. A more detailed description of Welch’s method is included in the Appendix A.

All feature extraction using the above data preprocessing pipelines was carried out prior to segmentation of the data into training, validation, and test sets for ML modeling and performance comparisons.

### 2.3. Modeling

Key procedural steps in the ML modeling process included the following:

1. The performance of four different ML models (SVM, RF, XGB, CNN) was assessed using standard open-source implementations [79,80,81]. Model training, testing, and validation were carried using a standard leave-one-out K-fold cross-validation procedure [36,82,83,84,85], in which the dataset was first randomly split into a training set (80%) and a test (20%) set. Validation datasets were then generated by further dividing the training dataset into K subsets, or “folds”, where each fold is a group of test subjects, and each of the K folds is used once as a validation dataset (“leave one out”) while the remaining K-1 folds are combined together as the training dataset. This procedure guarantees that every test subject will be in a validation set exactly once and in a training set K-1 times. The error estimate is averaged over all K trials to derive the performance of each model. As is common practice, we use K = 5 to balance bias and variance of test error estimates [85]. Cross-validation was applied multiple times for different values of the hyperparameters, and the parameters that optimized each model were selected by maximizing the concussion classification F1-score across each of the selected validation folds (F1 ± 2 standard deviations). In this manner, cross-validation addresses the problem of overfitting [82], since cross-validated models that perform well over the test data and give good accuracy have not overfitted the training data and can be used for prediction. The hyperparameters that optimized each model are listed in Appendix A.
2. The classification performance of the four different ML models was ranked based on the F1 scores when applied to the testing set. The F1-score represents a balanced approach for conveying a model’s performance in terms of its correct and incorrect classifications. Specifically, F1 weighs both false negatives (FN) and false positives (FP) in conveying a model’s accuracy and is prioritized for ranking the performance of the current ML models for both binary (Use Case 1) and multiclass (Use Case 2) classification experiments. All metric descriptions and formal calculations are provided in Appendix A.
  (1) $$F1=\frac{2}{\frac{1}{recall}+\frac{1}{precision}}=2\frac{precision*recall}{precision+recall}=\frac{tp\;}{tp+\;\frac{1}{2}\left(fp+fn\right)}$$
3. In Use Case 1, the random assignment of “healthy” and “concussed” individuals into training, validation, and testing datasets maintained the original proportional balance in each dataset.
4. In Use Case 2, the random assignment of the concussed individuals into training, validation, and testing datasets for multiclass prediction (“vestibular” vs. “neurological” vs. “both”) also maintained the original proportional balance in each dataset.

The present study did not further subdivide test subjects according to age or gender. As we previously reported [58], although there are small but measurable differences in phybrata performance between males and females and between younger and older participants in healthy test groups, these differences are completely masked by the much larger variations that result from concussion-induced balance disruptions and are thus not statistically relevant for the present diagnostic analyses.

## 3. Results

### 3.1. Use Case 1: Classifying “Healthy” vs. “Concussion”

Presented below are standard performance metrics for each combination of ML model and preprocessing pipeline used to classify the testing set (*n* = 34) into “concussion” vs. “healthy” groups. We primarily emphasize the F1 metric for comparing across conditions because it combines precision and specificity into a single unified score; specificity and sensitivity are also provided separately.

#### 3.1.1. Comparison of TSA and NTS Data Preprocessing Pipelines

Table 1 compares healthy vs. concussion classification metrics (F1, sensitivity, specificity) across all combinations of ML model and preprocessing pipeline. Overall, global features extracted from the phybrata signals using the NTS pipeline performed better than averaged time-steps in the TSA pipeline. Averaging across all models, NTS preprocessing led to an overall 10% improvement in F1 when compared to TSA preprocessing. RF emerged as the best model for both preprocessing conditions, but with no change in F1 (TSA = 0.94, NTS = 0.94), whereas SVM F1 increased by 29% (TSA = 0.62, NTS = 0.91), and XGB F1 increased 3% (TSA = 0.91, NTS = 0.94). The change in CNN performance between TSA and NTS preprocessing was not captured due to the architecture of the model strictly requiring a time-series input signal, therefore only the TSA pipeline performance is compared for CNN F1 (TSA = 0.91).

#### 3.1.2. Machine Learning Model Comparisons

Table 1 also allows a comparison of the overall classification performance of the four different ML models. Collapsing across TSA and NTS preprocessing and averaging each of the three metrics, RF outperformed all ML models for F1 (RF F1 = 0.94, SVM F1 = 0.77, XGB F1 = 0.93). RF and SVM achieved the highest specificity score (RF specificity = 0.91, SVM specificity = 0.91, XGB specificity = 0.88), whereas RF and XGB achieved the highest sensitivity score (RF sensitivity = 0.97, SVM sensitivity = 0.71, XGB sensitivity = 0.97). Compared within TSA preprocessing, RF and SVM achieved the highest specificity score, whereas RF and XGB achieved comparable sensitivity scores. Based upon the F1 scores for TSA preprocessing, RF was the stand-alone best model, including the neural network (CNN F1 = 0.91). For NTS preprocessing, RF and XGB were identical in their superior performance across all metrics (specificity = 0.88, sensitivity = 0.99, F1 = 0.94). The fact that the RF model performs well over the test data and gives good accuracy indicates that the validated model has not overfitted the training data and is a suitable candidate for further experimental prediction [82].

#### 3.1.3. Concussed vs. Healthy SHAP for RF NTS Model

**Figure 2:**
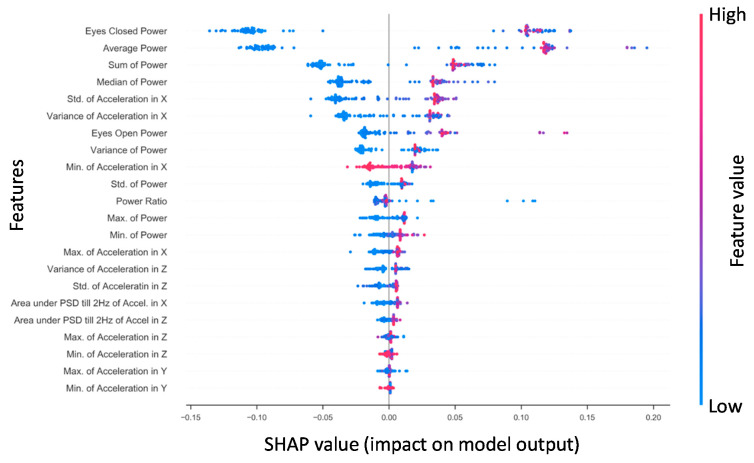
The Random Forest SHAP values for all patients in the training set (*n* = 108). Features are ranked top to bottom (top being the largest contributor). A single point represents a patient‘s SHAP value for a given feature. Along the *x*-axis, a positive (negative) SHAP value indicates the features’ impact toward classifying a patient as concussed (healthy control). Color indicates the actual value of a subject’s feature value.

Shapley additive explanations (SHAP) is a method for explaining how complex machine learning models make decisions with the data they receive by computing the magnitudes of the contributions from each individual feature to a model’s output [86]. Figure 2 presents the SHAP values for features derived from the *x*-axis phybrata signals of training set patients for the best performing RF model. Feature importance is ranked from top to bottom (left *y*-axis). The two most robust features in terms of their contributions to classification are the Ec power and average power, consistent with previous results [58]. The third and fourth most robust predictors were the sum and median power, which showed a similar pattern of values in relation to outcome. These power features are followed by the *x* acceleration features, particularly standard deviation and variance in the global *x* (A–P) signal. Notable features that were considered reasonably useful for RF training and prediction were eyes-open power, the variance of power, and the Ec/Eo power ratio. The *y* (vertical) and *z* (M–L) features were found to be less robust in prediction for RF. The actual values of the features (right *y*-axis) are suggestive that higher *x* (A–P) accelerations and phybrata powers (red) are linked with a prediction of concussion, while lower accelerations and powers (blue) and smaller *x* feature values are linked with a prediction of healthy, with the exception of the global minimum for *x* (Min. of Accel X) and *z* (Min. of Accel Z). Less significant *z* and *y* features for RF also appear clustered around the center axis at the bottom of the plot, indicating minimal predictive impact on model output.

### 3.2. Use Case 2: Concussion Impairment Classification

In Use Case 1, the binary classification (healthy vs. concussion) F1 performance for all ML models was highest using NTS preprocessing, except for RF where NTS and TSA FI performance was equivalent. In Use Case 2 we further assess the predictive utility of NTS preprocessing features when used with the three bestt performing ML models for the multiclass prediction of three specific concussion-related impairment states (vestibular impairment, neurological impairment, or both).

#### 3.2.1. Comparison of Model Performance

Table 2 summarizes the multiclass performance for the three NTS models: RF (specificity = 0.93; sensitivity = 0.89; F1 = 0.90); SVM (sensitivity = 0.83; specificity = 0.72; F1 = 0.73); and XGB (specificity = 0.93; sensitivity = 0.83; F1 = 0.85). RF was clearly the best performing model, showing superior performance across multiple performance metrics including a 5% improvement in F1 over XGB and 17% improvement in F1 over SVM.

**Figure 3:**
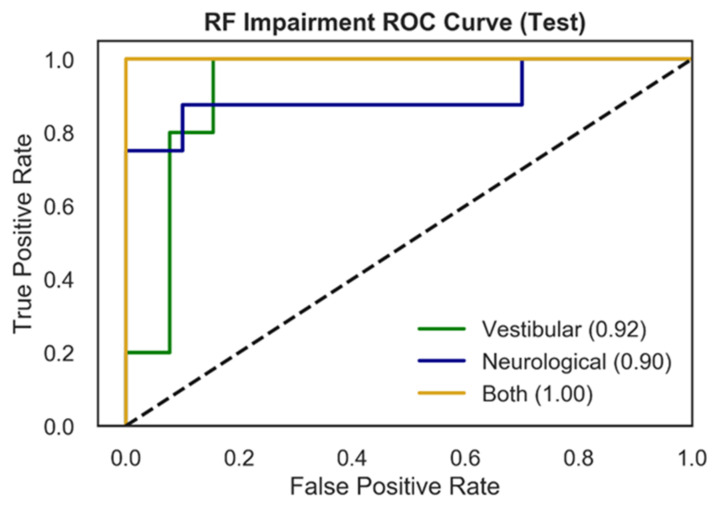
ROC curves for multiclass impairment prediction using the RF model and NTS preprocessing pipeline. Each curve represents the specific impairment vs. the rest of the classes (one vs. rest) and depicts the trade-off between True Positive Rates (TPR) and False Positive Rates (FPR) for each physiological impairment condition. The ROC performance is based upon the optimal threshold selected by the model for the testing set. The three colored curves correspond to vestibular impairment = green, neurological impairment = blue, both = yellow. The dotted black line represents random performance.

Figure 3 presents RF test ROC curves for the multiclass impairment prediction, with each curve representing the specific impairment vs. the other two diagnoses (vestibular vs. rest; neurological vs. rest; both vs. rest). ROC curves represent the tradeoff between the True Positive Rate (TPR) and False Positive Rate (FPR) for an optimized decision threshold. This decision threshold is learned during model training and translated to the testing set used to generate the curves in Figure 3. The area underneath each curve (AUC) in Figure 3 quantifies model performance in relation to TPR and FPR. Vestibular and neurological ROC curves showed AUC scores of 0.92, and 0.90 respectively, while the ROC curve for patients with both impairments exhibits an AUC of 1.00.

**Figure 4:**
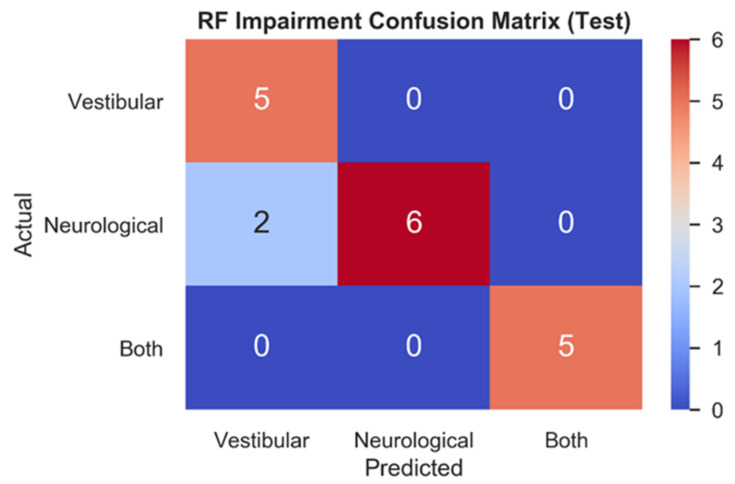
Testing set confusion matrix of RF model. Model predictions for each impairment (*x*-axis) are contrasted with actual impairment outcomes (*y*-axis) to categorize the correct and incorrect predictions made.

Figure 4 depicts the RF testing set confusion matrix (*n* = 18) for the three impairment classes. RF correctly classified all vestibular (1.0, 5/5) patients, most neurological (0.75, 6/8) patients, and all patients with both (1.0, 5/5) impairments. The two mistakes in the test set both relate to model predictions of vestibular impairments for patients who had been clinically diagnosed with neurological impairments.

#### 3.2.2. Specific Impairment SHAP for NTS RF Model

**Figure 5:**
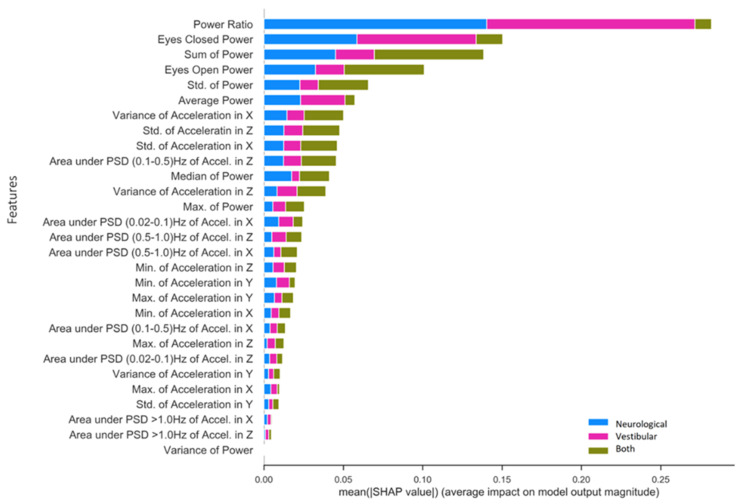
Mean SHAP value contribution (*x*-axis) of each feature (*y*-axis) for every impairment condition (blue = neurological, pink = vestibular, green = both), ranked from top to bottom in terms of importance. Taking each class together, the total mean SHAP value reflects each feature’s global impact on model classification.

Figure 5 presents SHAP value rankings of feature importance for the multiclass application of the RF model using the NTS preprocessing pipeline. The Ec/Eo power ratio was clearly the most robust predictor overall in the NTS RF model, contributing significantly to the classification of neurological (SHAP = 0.14) and vestibular (SHAP = 0.13) impairments as opposed to both (SHAP = 0.01). Ec and Eo power features made the second and fourth-largest contributions, and also captured unique aspects of variation in each impairment that differ from the Ec/Eo power ratio. In particular, Ec power contributed to neurological (SHAP = 0.05) and vestibular (SHAP = 0.08) impairment classifications, but very little for individuals with both (SHAP = 0.02). Conversely, Eo power and Sum Power account for individuals with both (Eo SHAP = 0.05; Sum power SHAP = 0.06) significantly better than Ec power, slightly less for neurological (Eo SHAP = 0.03; Sum power SHAP = 0.05) and significantly less for vestibular (Eo = 0.02; Sum power SHAP = 0.02) impairments. Finally, standard deviation of power and Average Power (Ec + Eo)/two rounded out the top six features for predicting neurological impairment (Std. power SHAP = 0.02; Average power SHAP = 0.02), vestibular impairment (Std. power SHAP = 0.01; Average power SHAP = 0.03) and both impairments (Std. power SHAP = 0.03; Average power SHAP = 0.01). The highest-ranking phybrata frequency band feature is the “*Area under PSD (0.1–0.5) Hz of Accel. in z*”, which is the frequency band corresponding to vestibular regulation of postural control. It is important to note here that the relative contribution of various phybrata features to specific impairments may be related to the ML model as well as the data consumed by the model, so that the SHAP value rankings shown in Figure 5 may change for different ML models.

## 4. Discussion

Identifying an accurate, reliable, and easy-to-use physiological impairment predictor for concussion injuries has been a long-standing pursuit in traumatic brain injury research and sports medicine. The present study demonstrates the first combination of a wearable motion sensor and machine learning model that enable the classification of both concussion injuries and specific concussion-related neurophysiological impairments. For Use Case 1, Table 3 compares the present results to other published results for ML-based binary classification of concussion patients vs. healthy control populations using a variety of other data sources. The combination of phybrata sensing and ML delivers overall performance (sensitivity, specificity, F1, AUC) exceeding these previously reported alternative approaches to ML-based concussion diagnostics.

The performance observed for Use Case 1 (Table 1, Figure 2, Table 3) demonstrates that the NTS RF model is well suited to identifying features in phybrata signals that distinguish between healthy individuals and those diagnosed with concussion injuries. This result is significant because the lower computational complexity of the RF model, compared for example to CNN models, may allow impairment classification to be incorporated directly into the phybrata sensor. Such a wearable device, capable of cloud-independent classification and quantification of multiple physiological impairments, would greatly enhance remote patient monitoring and management of many chronic medical conditions. RF models are also of interest because they are robust to overfitting and can produce competitive results compared to more computationally complex algorithms [87].

One limitation of the current work is that the sample size for comparing patient groups was relatively small (*n* = 169). This can present uncertainty for several components of the ML process including training, hyperparameter and model selection, and evaluating model performance. However, the effect sizes previously reported for the current study population [58] can be used to calculate the predictive power expected from this sample size [88]. For example, the mean and standard deviation (SD) values for the Average Phybrata Power (Eo + Ec)/two for the baseline and concussion populations are (i) Baseline: sample size = 83, Mean = 0.355, SD = 0.104; (ii) Concussion: sample size = 92, Mean = 1.348, SD = 0.955. Based on this effect size, the present study cohort should allow a predictive power greater than 0.90 using an α value of 0.05. This high expected predictive power, together with the consistent performance observed throughout ML training, testing, and validation, indicate that the present results present a valid assessment of the classification performance of the ML models that we investigated in combination with the phybrata sensor data.

Consistent with our previous studies [58], the present results also demonstrate that Eo and Ec phybrata power measurements deliver robust metrics for differentiating between healthy and concussed individuals. The phybrata power provides a composite metric for phybrata motion along the *x*, *y* and *z* axes, all of which make distinct contributions to the power signal. The NTS RF SHAP values suggest that higher phybrata power values are indicative of concussions regardless of Eo or Ec phase, whereas lower phybrata power reflects healthy functioning. This result is consistent with the observation that the postural instabilities induced by head impact injuries lead to easily observed increases in phybrata-related motion of the body. The RF model results indicate that elevated levels of phybrata-related motion along all three (*x*, *y*, *z*) axes accompany concussion injuries, with contributions that can arise from impairments to multiple physiological systems and related sensory reweighting [58].

Our previous ROC analyses [58] indicated that the average power (Eo + Ec)/2 has the best diagnostic performance for clinical diagnosis of concussion, while Eo and Ec/Eo have the best diagnostic performance as independent measures to confirm accompanying neurological and vestibular impairments, respectively. All three measures demonstrated AUC, sensitivity, specificity, and accuracy above 90% for their respective diagnoses. In the present ML analyses, the NTS RF results confirm the predictive utility of Ec power and average power in differentiating healthy individuals from concussion patients. In the context of identifying specific concussion impairments, the present results also confirm that the phybrata power ratio (Ec/Eo) is highly correlated with phybrata fluctuations caused by neurological and vestibular impairments. The Ec/Eo power ratio was clearly the most robust predictor for separate neurological and vestibular impairments, while the sum power and Eo power most significantly accounted for individuals with both impairments.

Our SHAP results differ slightly from previous statistical analyses [58] in that the Ec/Eo ratio most significantly accounts for separate neurological and vestibular impairments, whereas Eo power explains individuals with both impairments most strongly. This means that the NTS RF model utilizes features derived using both the Eo and Ec phybrata powers to gain relevant predictive insight into multiple physiological impairment conditions. In this context, it is important to note that SHAP feature ranking is a model-sensitive framework for understanding the contribution of various features to ML model predictions. Different models may leverage different features and feature sets to generate predictions about each impairment, which is expected to lead to variability in the identification and ranking of the specific phybrata features that contribute to the classification of different physiological impairments. The present results for Use Case 2 (Table 2, Figure 5) highlight the utility of NTS RF for not only characterizing dysfunction but delivering highly precise multiclass predictions (F1 = 0.90). As we previously reported, Eo and Ec phybrata features were differentially predictive of clinical impairment [58], and the present NTS RF model results confirm this past work. The one published machine learning study relevant to Use Case 2 carried out a retrospective cluster analysis on the balance and vestibular diagnostic data of concussion patients using two different clustering tools (K-means and self-organizing map) [37], and demonstrated the presence of two distinct groups, one with prominent vestibular disorders and another with no clear vestibular or balance problems. However, this study did not include ROC analyses of diagnostic sensitivity, specificity, F1, or AUC.

An important result of the present study is that, compared to our previously reported manual feature inspection and statistical data analysis [58], the RF model was able to identify a wider range of relevant phybrata signal features that contributed to impairment classification, and achieved similar diagnostic performance. This result indicates that the large set of time domain, spatial domain, and frequency domain features and distinct frequency bands [64,65,66,67,68] that reflect the contributions of different physiological systems to balance and postural stability can be mined as a rich source of information for understanding concussion pathology and delivering strong diagnostic performance using machine learning-based approaches. For example, as illustrated in Figure 5, several phybrata PSD frequency bands (including the band corresponding to vestibular regulation of postural control) contribute to multiclass impairment predictions in the concussion patient cohort. As we previously reported [58], phybrata PSD plots reveal the sensory reweighting across multiple physiological systems that is triggered by impairment to any single physiological system. Such composite sensory reweighting features, based on correlated PSD variations across multiple phybrata spectral bands, may enable the derivation of phybrata biomarkers that enable more sensitive, earlier, and more specific diagnoses of neurological conditions based on unique impairment signatures. This will be a topic of future investigations.

The present results indicate that phybrata measurements in both Eo and Ec test phases, as well as derivative measures such as their average, ratio, and sum, are important for capturing variability in patients with different physiological system impairments and should be utilized when assembling a profile of patient dysfunction. Further work needs to explore vestibular impairment in greater detail as it is an important neurosensory system serving diverse functions [89,90]. Vestibular impairments accompany many neurological conditions and can lead to widespread disruptions throughout the central nervous system [71,72,73]. For this reason, a major focus has been on general mechanisms of recovery, repair, and compensation in associated neural circuits and functional processing regions [91]. The manner in which specific physiological disruption mechanisms are reflected in phybrata measurements is now being studied for many other neurological conditions in addition to concussions.

The static balance and postural stability testing capabilities of the phybrata sensor also have recently been expanded to include locomotion by developing on-body sensor calibration and phybrata implementations of widely used dynamic motion tests such as Activities of Daily Life (ADL) and Timed-Up-and-Go (TUG) [61]. A single head-mounted phybrata sensor was shown to enable classification and quantification of complex gait parameters with performance matching current gold standard solutions that require expensive clinical video motion capture systems [61]. Combining static balance and dynamic gait testing significantly expands the available set of observable phybrata features that can be used in the derivation of impairment and disease biomarkers. Mapping this rich set of phybrata features to impairment severity and recovery trajectories may provide important insights into tailoring treatment and rehabilitation for many different neurological conditions that are accompanied by balance and locomotion disruptions, including concussions, stroke, multiple sclerosis, Parkinson’s disease, elderly frailty, and peripheral neuropathies. This wide range of potential applications makes it even more important to leverage machine learning for feature identification and importance ranking, and places priority on low-computational-complexity ML models that can be integrated into wearable devices.

It is also important to consider that impairments to the vestibular, neurological, and other physiological systems will trigger changes in phybrata signals that can depend on a range of factors that include the nature and severity of the injury, the developmental age of the patient [23,92], and the phybrata testing phase (Eo vs. Ec) [58,69]. Further explorations of the influence of these factors on the rich set of observable phybrata features may provide deeper insights into individual impairment pathologies and potential routes to further enhance the diagnostic capabilities of ML algorithms in combination with phybrata data.

## 5. Conclusions

In this study, we investigated the performance of four ML models (SVM, RF, XGB, CNN) used to refine the neurophysiological impairment assessment capabilities of phybrata wearable sensors, utilizing data from a previously studied concussion patient cohort. Two distinct data preprocessing pipelines (TSA, NTS) were first investigated for binary classification of the test population as heathy vs. concussion. The NTS model approach was found to outperform the TSA approach, with the best algorithms achieving an F1 score of 0.94. Next, the three best-performing NTS models were compared in terms of their multiclass prediction performance for specific concussion-related impairments (vestibular, neurological, both). The NTS RF model achieved the best classification performance in the testing set with an F1 score of 0.90. ML models identified a wider range of relevant phybrata signal features that contributed to impairment classification and achieved diagnostic performance similar to previously reported manual feature inspection and statistical data analysis. This wider range included phybrata PSD spectral band features corresponding to specific physiological system contributions to postural control, which may enable the derivation of composite sensory-reweighting biomarkers that enable more sensitive, earlier, and more specific diagnoses of neurological conditions based on unique impairment signatures. The overall classification performance achieved in the present work exceeds previously reported approaches to ML-based concussion diagnostics.

The present results demonstrate that lower computational complexity ML models such as NTS RF are well suited to identifying features in phybrata signals that distinguish between healthy individuals and those with concussion injuries, and can further identify the presence of specific underlying neurological and vestibular impairments. The marriage of phybrata and ML holds significant predictive, diagnostic, and prognostic potential at scale and presents important new opportunities for understanding the pathology and progression of complex neurological conditions, as well as remote monitoring and proactive patient management in a wide range of medical, athletic, military and consumer applications.

## 6. Patents

USPTO Provisional patent application 63/113,917, filed 11/15/2020.

Physiological impairment biomarkers and detection methods.

## Figures and Tables

**Table 1:** Classification performance (sensitivity, specificity, F1) of four different ML models and two different data preprocessing pipelines for binary prediction of “healthy” vs. “concussion”. The testing dataset (*n* = 34) classes were representative of the training set distribution (Healthy = 17, Concussed = 17).

Use-Case	Model	Preprocessing Pipeline	Specificity	Sensitivity	F1
Concussed vs. Healthy	RF	TSA	0.94	0.94	0.94
		NTS	0.88	0.99	0.94
	SVM	TSA	0.94	0.47	0.62
		NTS	0.88	0.94	0.91
	XGB	TSA	0.88	0.94	0.91
		NTS	0.88	0.99	0.94
	CNN	TSA	0.88	0.94	0.91

**Table 2:** Classification performance (sensitivity, specificity, F1) of three different ML models using the NTS data preprocessing pipeline for multiclass prediction of concussion-related impairments. The testing dataset (*n* = 18) classes were representative of the training set distribution.

Use-Case	Model	Preprocessing Pipeline	Specificity	Sensitivity	F1
Vestibular vs. Neurological vs. Both	RF	NTS	0.93	0.89	0.90
	SVM	NTS	0.83	0.72	0.73
	XGB	NTS	0.93	0.83	0.85

**Table 3:** Comparison of results for binary classification of concussion patients (nr = not reported).

Data Source	ML Model(s)	Sensitivity	Specificity	F1	AUC	Reference
Phybrata sensor	RF, SVM, XGB, CNN	0.94	0.94	0.94	0.98	present work
Multimodal: Neurocognitive tests, clinical scales, symptoms checklists, balance and gait testing	CNN	nr	nr	0.85	0.95	[30]
MRI	SVM	0.89	0.79	0.84	0.84	[31]
Multimodal: EEG, neurocognitive tests, standard concussion assessment tools	Genetic Algorithm (GA) classifier	0.92	0.75	0.81	0.92	[32]
EEG	SVM	0.82	0.80	0.81	nr	[34]
EEG	Genetic Algorithm (GA) classifier	0.98	0.60	nr	0.90	[36]
Clinical scales and assessment metrics: retrospective analysis	C5.0 Decision Tree, Recursive Partitioning, Random Forest, XGB	0.97–0.99	0.43–0.58	0.71–0.78	nr	[38]
Eye tracking	CNN	0.63	0.74	0.67	0.75	[41]
3 blood biomarkers	Random Forest	0.98	0.72	nr	0.91	[42]
Head impact data	SVM, Random Forest, CNN	0.84	0.88	0.86	0.9	[45]

## Data Availability

Data available on request due to restrictions e.g., privacy or ethical. The data presented in this study are available on request from the corresponding author. The data are not publicly available due to data disclosure restrictions of the organizations participating in this study.

## Appendix A

Section 2.2 Data Preprocessing and Comparison of ML Models

Machine Learning Model Information

The Support-Vector Machine and Random Forest classifiers described in this work are both drawn from the open-source machine learning package scikit-learn (https://github.com/scikit-learn/scikit-learn (accessed on 16 June 2020)). The gradient boost classifier (XGBoost) was also taken from an open-source framework (https://github.com/dmlc/xgboost (accessed on 16 June 2020)). Finally, the convolutional neural network described in this work was constructed using an established open-source deep learning framework known as PyTorch (https://github.com/pytorch/pytorch (accessed on 16 June 2020)). The hyperparameters used for each optimized model are listed below.

Use Case 1: Binary Classification of Concussion

Non Time-Series (NTS) Data Preprocessing

Support Vector Machine (SVC)

- C = 20
- decision_function_shape = “ovr”
- kernel = “rbf”
- degree = 3
- probability = True
- class_weight = “balanced”

Random Forest Classifier (RF)

- oob_score = True
- n_estimators = 150
- class_weight = “balanced”
- min_samples_leaf = 2
- criterion = “gini”
- max_depth = None
- max_features = squareroot(n_features)

XGBoost (XGB)

- n_estimators = 15
- max_depth = 6
- gamma = 0
- learning_rate = 0.3
- min_child_weight = 1
- subsample = 1

Time-Series Averaging (TSA) Data Preprocessing

Support Vector Machine (SVC)

- C = 15
- decision_function_shape = “ovr”
- kernel = “rbf”
- degree = 5
- probability = True
- class_weight = “balanced”

Random Forest Classifier (RF)

- oob_score = True
- n_estimators = 150
- class_weight = “balanced”
- min_samples_leaf = 2
- criterion = “gini”
- max_depth = None
- max_features = squareroot(n_features)

XGBoost (XGB)

- n_estimators = 50
- max_depth = 8
- gamma = 0
- learning_rate = 0.4
- min_child_weight = 1
- subsample = 1

Convolutional Neural Network (CNN)

Convolutional Layer

- input_channels = 4
- output_channels = 64
- kernel_size = 6
- stride = 1
- activation_function = “ReLU”

Convolutional Layer

- input_channels = 64
- output_channels = 128
- kernel_size = 6
- stride = 2
- activation_function = “ReLU”

Linear Layer

- input_channels = 640
- output_channels = 100
- activation_layer = “ReLU”
- Linear Layer
- input_channels = 100
- output_channels = 1

Use Case 2: Multiclass Prediction of Concussion Impairments

Non-Time-Series (NTS) Data Preprocessing

Hyperparameters are identical to NTS models in Use Case 1

Time-Series Averaging (TSA) Data Preprocessing

For each Eo and Ec patient test, the three phybrata time-series signals (*x*, *y*, *z*) and the phybrata power (calculated using the vector sum of the three acceleration components) were averaged over one-second time-steps (100 samples per time step t), generating twenty averaged time-steps (20*t*) for each 20-s patient test. In the SVM, RF, and XGB ML models, every *t* becomes a feature (*d*) for each of the accelerometry predictors (*d*; power, *x*, *y*, *z*) forming an *n* × (20*t* ∗ 4*d*) matrix for each of the Eo and Ec conditions and n patients. The CNN model architecture requires that the input data be formatted as an (*n* ∗ 20*t*) × 4*d* matrix.

Phybrata Power Calculations

Phybrata power is calculated as follows:

p = *m_B_* ∗ a^2^ ∗ *t*

*m_B_* is the average mass of the human brain (*m_B_* = 1.2 kg). For each individual point in time, the RMS acceleration is calculated as (a*_x_*^2^ + a*_y_*^2^ + a*_z_*^2^)^1/2^. The phybrata power is calculated for each time sample duration ∆*t* (∆*t* = 0.01 s for 100 Hz sampling rate) and then summed across the full 20-s time series for each of the Ec and Eo test phases.

Non-Time-Series (NTS) Feature Extraction—Phybrata Signal PSD Calculations

Welch’s method [78] splits a time-series into overlapping segments, computes periodograms for each segment using a discrete Fourier Transform, squares the results to generate a power spectral density (PSD) curve for each periodogram, and then averages all the results to generate a single smoothed PSD curve that is representative of the entire time-series signal. This PSD curve can then be used to calculate phybrata signal powers within specific frequency bands. The Welch’s method calculations in the present work used the following parameters for each of the time-series signals: total number of samples = 2000 (20 s signals with a sampling rate of 100 samples/segment); segment length = 100 samples; segment overlap = 50%; total number of segments = 40.

ROC Calculations

The following abbreviations are used:

TP = True Positives; TN = True Negatives; FP = False Positive; FN = False Negative.

Formulas for specific evaluation metrics:

Sensitivity = (TP)/(TP + FN)

Specificity = (TN)/(TN + FP)

F1 = (TP)/(TP + (½ × (FP + FN)))
